# Advancing Arbovirus Research in the Caribbean and Latin America: 2025 Global Virus Network Regional Meeting

**DOI:** 10.3390/v17101330

**Published:** 2025-09-30

**Authors:** Tiffany R. Butterfield, Joshua J. Anzinger, John Lindo, Gene D. Morse, Sten H. Vermund, Maggie L. Bartlett

**Affiliations:** 1 Global Virus Network, Tampa, FL 33612, USAsvermund@gvn.org (S.H.V.); 2 Department of Microbiology, University of the West Indies, Mona, Kingston 7, Jamaica; 3 Center for Integrated Global Biomedical Sciences, University at Buffalo, New York, NY 14214, USA; 4 College of Public Health, University of South Florida, Tampa, FL 33620, USA; 5 Bloomberg School of Public Health, Johns Hopkins University, Baltimore, MD 21205, USA

**Keywords:** virology, arboviruses, pandemic preparedness

## Abstract

A May 2025 symposium convened leading virology experts across Latin America and the Caribbean (LAC) to advance regional research and collaborative efforts. Sessions explored cutting-edge developments in arbovirology, pressing challenges in viral surveillance, and the complexities of vector biology. Integrated networking opportunities and hands-on workshops offered mentorship and training, focused on the next generation of virologists, and strengthened scientific communication within the region. The morning session included reports from the LAC Global Virus Network (GVN) Centers of Excellence. A roundtable dialogue tackled the present challenges faced in arbovirus research. The Abbott Pandemic Defense Coalition reported on its collaborative progress. Trainees from the University at Buffalo, the State University of New York, and the University of the West Indies Global Infectious Diseases Research Training program showcased their current research projects. A session concentrated on health landscapes and the capacity for viral vaccinations within the region. A mentoring workshop focused on immune evasion methodologies and obstacles associated with arboviruses. One Health perspectives on viral zoonotic diseases addressed developments in the surveillance of vector-borne viruses in the Caribbean. Studies of mosquitoes and ticks as vectors of viruses included discussion on the neurovirulence of arboviruses and symptoms occurring after viral infections. Pediatric infectious diseases were highlighted in their environmental health context. An additional mentoring workshop centered on viruses and the microbiome. The relationship between viruses and cancer was discussed in the South American context and included recent advancements in the field of vaccinology. The Jamaica Regional GVN meeting promoted collaboration, facilitated the exchange of knowledge, and advanced research efforts throughout the region.

## 1. Overview

**Figure 1:**
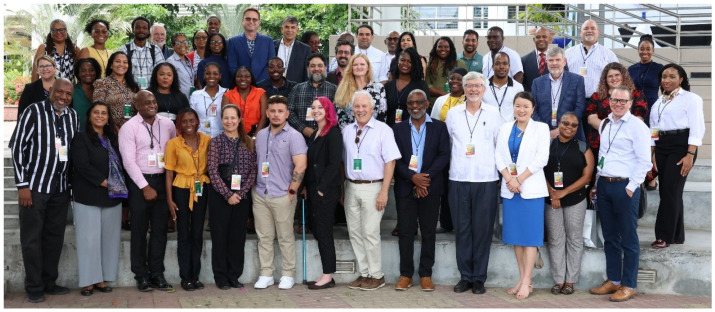
Fifty of the participants of the 2025 Global Virus Network Regional Meeting.

Dr. John Lindo (Professor at the University of the West Indies (UWI), Mona Campus) opened the meeting in Jamaica and underscored the importance of regional meetings of experts and their importance to advancing pandemic preparedness globally (Figure 1). Dr. Sten Vermund (Dean of the University of South Florida College of Public Health and GVN Chief Medical Officer) introduced the new GVN initiative to categorize expertise and capacity across Central America, South America, and the Caribbean to improve pandemic preparedness by connecting Latin America and Caribbean (LAC) experts to regional U.S. Centers for Disease Control and Prevention (CDC) offices. Dr. Vermund noted the recent substantial loss of support to global research from U.S. federal funds and called for investment from other sectors to ensure that critical LAC investments are sustained. One example is the specimen repository at the University of Texas Medical Branch (UTMB), a GVN Center of Excellence (CoE), that has lost significant funding after 60 years of continuous funding; this repository has aided in innumerable research projects that benefited the U.S. and the world [1].

## 2. GVN Regional Center of Excellence and Affiliate Updates

Dr. Calum MacPherson (Professor at St. George’s University and GVN Affiliate Director) gave an overview of his and his colleagues’ work, encompassing bats as viral vectors, viruses of sea turtles, mongoose rabies, and arboviruses including Zika, chikungunya, and dengue [2,3]. He underscored that the cause of approximately 80% of acute febrile illness cases in Grenada is unknown, with the other 20% being attributable to the dengue virus, and the need for better diagnostics for other causes. Dr. Eduardo Gotuzzo (Professor at the Peruvian Cayetano Heredia University Institute of Tropical Medicine “Alexander von Humboldt” and GVN CoE Director) presented the current understanding of HTLV-1 and co-infections in LAC [4,5,6,7]. Dr. Gotuzzo further highlighted the need for funding to better characterize the relationship between HTLV-1 and cancers. Dr. Joshua Anzinger (Senior Lecturer and Head of Department for UWI-Mona and GVN Affiliate Director) reviewed the utility of Jamaica as a hub for pathogen surveillance and the prevalence of dengue virus serotype 3 regionally [8,9]. Interestingly, Jamaica’s rates of HTLV-1 prevalence have remained steady at 2% over the last 20+ years; however, due to a lack of routine screening, there are likely asymptomatic cases that could be detected earlier to enhance recognition of cancers and prevention of vertical transmission [10,11]. Dr. Rubens Alves (Principal Investigator at the Institut Pasteur de São Paulo) emphasized the need for One Health as a focus, using Brazil’s ecosystem as a model. The keys to leveraging this include complementary expertise, capitalizing on Brazil’s biodiversity, and positioning South America as a Global Health Hub [12].

## 3. Abbott Pandemic Defense Coalition Update

Dr. Lester Perez (Principal Scientist at Abbott) updated the attendees on the outcomes of the Abbott Pandemic Defense Coalition (APDC) initiative [13]. Dr. Perez underscored that time is of the essence when a pathogen emerges, and APDC has grown to include 20+ countries and 5 continents, including GVN CoE’s UTMB and University at Buffalo, State University of New York. Through April 2025, the APDC has provided 690,396 on-market tests and 24,164 research tests to partner sites, developed 48 new prototype tests, collected 36,758 specimens, identified 6 significant outbreaks in 3 countries, trained 116 future virus hunters through FETP, published 108 peer-reviewed papers, and identified 24 new viruses [14,15,16,17,18,19]. The Yellow Fever Virus (YFV) causes 200,000 cases annually, with 60,000 deaths on average, with a range from asymptomatic to severe disease presentations in Colombia [20]. From 2020 to 2023, 53.3% of cases of acute febrile illness (*n* = 2528) were not dengue or malaria, and 52 of them were sequenced, resulting in the identification of a new strain of YFV. Analyses suggest that the emergence of the Colombia/Bolivia clade resulted from episodic positive selection in non-structural protein 2 a (NS2a). This may indicate a benefit to transmissibility, though more research is needed to further elucidate this observation. Most YFV strains circulating in Colombia originated from the country itself, suggestive of cryptic circulation [20]. Although Ecuadorian colleagues were not in attendance, there are analogous challenges in that neighboring nation [21,22,23].

## 4. Global Infectious Disease (GID) Research Training Program

Dr. John Lindo and Dr. Gene Morse (State University of New York (SUNY) Distinguished Professor at the University at Buffalo HIV and HCV Clinical Pharmacology Laboratory and GVN CoE Director) underscored the need for programs like these to train the next generation. Trainee projects encompassed diverse aspects of virology research, including studies on the clinical outcomes of viral infections, viral surveillance in human populations and vectors, as well as the application of cloud-based computing and molecular epidemiology. Samatha Mosha Miller (pediatric resident at UWI-Mona) described her work on long COVID in pediatric cases. Alton Bodley (PhD candidate at UWI-Mona) discussed his work on cloud-based computing and viral surveillance in Jamaica. Within Jamaica, sentinel sites include hospitals, health centers, and hotels; they serve as the first line of detection and investigation. Once sentinel sites detect a pathogen, they are confirmed at UWI or one of the other designated labs. Public health action then occurs via the Ministry of Health Surveillance Unit. Alton developed a data lake with analytics in a dynamic collaborative data ecosystem for West Nile Virus (WNV), as the data were siloed and lacked metadata, such as monthly cases, climate at that time, and other key variables. Dr. Gene Morse emphasized that virology requires a multidisciplinary team, and mentored training grants have been the primary way we advance the next generation, especially trainees from low- and middle-income countries (LMICs). “Brain-drain” has always been a problem, sometimes inadvertently exacerbated by training visits. New approaches are needed from academic collaborations with government and industry partnerships to support LMIC scientists in their home nations. A current challenge is that there is a lack of commercial partners for sustaining these initiatives in LMICs based on prior investments and training.

## 5. Latin America as a Hub for Vaccine Research

Dr. Arlene Calvo (Associate Professor at USF and member of the Instituto de Investigaciones Científicas y Servicios de Alta Tecnología de Panamá (INDICASAT)) highlighted the importance of the trust between the public and public health. She shared information on the implementation of the pertussis vaccine in Panamá, which had an absolute vaccine effectiveness of 99.3% after three doses [24,25,26,27,28]. This meeting also included Continuing Medical Education workshops and mentoring opportunities focused on virus discovery and surveillance, arbovirology, challenges to training the next generation, and zoonotic viruses like dengue virus (DENV) in Grenada. Dr. Sten Vermund presented a review of new frontiers in vaccinology. Vaccination against measles, pertussis, mumps, rubella, smallpox, diphtheria, human papillomavirus (HPV), hepatitis B virus (HBV), hepatitis A virus (HAV), and poliovirus decreased morbidity from each by 97–100% by 2023 [29]. While protection from infection is rare (i.e., sterilizing immunity), protection from severe disease (symptomatic infection, hospitalization, and death) is universal for so-called vaccine-preventable diseases. Rapidly emerging vaccination technologies include new whole-organism vaccines, purified macromolecules, recombinant-vector vaccines, mRNA-based vaccines, and DNA-based vaccines. These applications are being expanded from preventing severe disease from infections to preventing the progression of cancer [30]. One vaccine technology may not be ideal for all pathogens, so multiple platforms should be explored to identify the best tool for each infection.

## 6. Re-Emerging Arboviruses

Dr. Scott Weaver (John Sealy Distinguished University Chair in Human Infections and Immunity, Professor at UTMB, and GVN CoE Director) shared that this decade has seen a 96% increase in DENV cases compared to the previous decade, highlighting the growing risk this virus poses. The Butantan-DV vaccine prevented DENV-1 and DENV-2 regardless of serostatus through a 2-year follow-up, and early evidence suggests quadrivalent protection. Dr. Weaver highlighted that there is not enough surveillance for arboviruses, but that the Brazilian efforts to expand epidemiological studies have helped identify a 2022 chikungunya virus (CHIKV) outbreak [31]. Another arbovirus, western equine encephalitis virus (WEEV), disappeared for many decades after a 1988 outbreak in Argentina and re-emerged in 2024. Exploration of submergence and re-emergence of a virus is critical to public health forecasting [32]. WEEV lost fitness for mammals but not for enzootic hosts like house sparrows; this may have happened by chance or may be due to unknown factors [33]. Oropouche virus (OROV) causes 11% acute febrile illnesses in Colombia, primarily transmitted by midges and can be transmitted by mosquitoes, sloths, and through human-to-human sexual transmission [34]. OROV has been hidden below the known and frequent dengue cases; diagnosis is low due to a lack of clinical diagnostics, but it needs study as a re-emerging arboviral threat.

## 7. Field Studies of Mosquitoes

Dr. Simone Sandiford (Lecturer at UWI-Mona) highlighted the need for field studies of mosquitoes. Dr. Sandiford’s team uses BG sentinel traps, aspirators, and resting shelters to collect mosquitoes from field locations to monitor arboviruses in Jamaica, in and around domiciles. Several niches exist, from urban to rural and wetlands to forested regions in Jamaica. Her team and collaborators generated the first complete *Aedes vittatus* mitochondrial genome, which is a critical reference for arbovirus research [35]. These data suggest that there have been multiple introductions of *Ae. vittatus* to Jamaica, and that this species has been present for some time. However, we often do not know where mosquitoes are located, what arboviruses are circulating, or which invasive species are present, underscoring the need to perform more field studies in Jamaica. Reintroduction of malaria to Jamaica and the struggle to eliminate it in 2006–2009 after a 44-year malaria-free period is a reminder of the vulnerability of tropical nations when vector-transmitted organisms are reintroduced [36,37].

## 8. Tick-Borne Viruses

Dr. Saravanan Thangamani (SUNY Empire Innovation Professor at SUNY Upstate Medical University) illuminated the role ticks and tick-borne viruses play in Jamaica [38]. Little is known about both the vectors and pathogens in the Caribbean, which is a significant gap in knowledge. Tick-borne viruses are accidental pathogens to humans, as 90% of ticks have preferred species specificity; however, the ones that do cause human disease cause significant mortality, as with the Crimean–Congo Hemorrhagic Fever virus. Ticks are competent vectors as they are persistent and slow feeders that can feed on a host for up to two weeks. Some tick species can live up to 20 years without a blood meal and lay as many as 18,000 eggs. Within the U.S., Heartland virus, Powassan virus (POWV), deer tick virus, Colorado tick fever virus, and Bourbon virus are concerns, but contribute to under 100 cases on average annually in humans. However, there are likely more cases, including those that are asymptomatic, but surveillance is lacking to determine the true epidemiology and disease risk. Global warming will lengthen tick seasons and expand geographic habitats, prolonging and extending the risk to humans of tick-borne diseases. POWV and deer tick virus cause distinct clinical outcomes and brain pathology [39]. There have been 340 reported human cases of POWV since 2004, with 44 deaths and 267 with neuroinvasive disease sequelae, and there has been a four-fold rise in cases from 2014 to 2023 compared to 2004 to 2013 [40]. In the U.S., POWV cases have increased from northeast to southwest following the distribution of the known vector that is across the entirety of the mid-U.S. to the east coast. To address the issue of collection for surveillance, the Upstate Tick Testing Laboratory was developed in New York State, U.S., to empower citizen science. Individuals can fill out a form if bitten by a tick and send the tick to the lab for the species to be identified, then nucleic acid is extracted and screened for 16 pathogens. Data is shared with the individual and presented in tickMAP [41]. Ticks carry more than one pathogen at one time, which is often overlooked in the one-pathogen one-vector mindset.

## 9. Dengue in Florida

Dr. Kristi Miley (Research Associate faculty at USF) described the globalization of disease risk through competent mosquito vectors with a focus on the dengue virus in Florida. From 2019 to 2024, there has been a 4× increase in dengue cases in Florida, similar to information presented by other speakers that highlighted Jamaica. These cases are primarily serotype 3, with 6:1 travel to local cases [42]. As climate shifts and salinity changes, the primary vector is expanding north, allowing for more human cases in more counties. Surveillance is critical but underperformed and requires more community involvement to address the gaps that exist. Further planning is needed to prevent outbreaks following hurricanes. Severe winds may reduce vector densities radically over the short run where wind velocities are great. However, longer-term increases in breeding sites or flooding where wind velocities are less severe can increase mosquito densities [43,44,45].

## 10. Microbiome

Dr. Christian Bréchot (Director of the USF Microbiomes Institute and Vice Chair of the GVN Board of Directors) illuminated the role viruses play in the microbiome and the need for further research, particularly across the emergence of new microbiome centers globally [46]. Integrating microbiome science into virology holds particular promise, as mounting evidence underscores the microbiome’s influence across health and disease. The human phenotype is shaped not only by the human genome but also by diverse microbial populations, particularly within the gut, where mucus layers and bacterial communities play protective roles. Disruptions in this microbial balance, known as dysbiosis, are increasingly linked to various diseases. Virus–microbiome interactions have profound implications, influencing susceptibility to infection, disease severity, vaccine response, and treatment outcomes, with compelling evidence across pathogens such as HIV, HPV, HBV, influenza, and SARS-CoV-2 [47]. For example, in COVID-19, characteristics of the gut microbiome have been associated with illness severity, suggesting potential for microbiome-targeted interventions [48,49]. Yet, current microbiome research faces limitations, including methodological inconsistencies, a lack of mechanistic studies, and overreliance on cross-sectional data. In audience discussions, the importance of virome research and the accessibility of oral microbiome studies were highlighted, with calls for comparative work across body sites and organisms, with further investment in studies of microbiomes from both humans and vectors.

## 11. Proactive One Health

Dr. Jean Paul Carrera (Investigator at the Instituto Conmemorativo Gorgas de Estudios de la Salud (Gorgas Memorial Laboratory) in Panamá) endorsed the need for active One Health surveillance of viruses, vectors, and reservoirs. His team set up mosquito traps with different hosts, including chicken, frog, hamster, and mice, and collected 11,000+ mosquitoes from Darien, Panamá. Within these vectors, numerous viruses were detected circulating, including Madrid, Aruza, Aguas Calientes, and Matusgarii orthobunyaviruses. Future work includes monitoring enrolled participants living in the region for seroconversion as well as febrile cases that present to nearby healthcare facilities. This work detected three previously undescribed orthobunyaviruses. Dr. Helena Solo-Gabriele (Professor at the University of Miami) discussed the application of wastewater surveillance (WWS) and novel methods to enhance detection, monitoring, and mitigation [50,51,52,53]. Her team has deployed WWS to detect SARS-CoV-2 in the community up to two weeks ahead of a surge and has shown utility in Miami for the detection of influenza A and B, poliovirus, respiratory syncytial virus, norovirus, and monkeypox virus [50,52,53]. This work is ongoing and will be expanded to detect arthropod-borne pathogens from dengue virus to malaria.

## 12. Excellence in Regional Leadership Award

Dr. Christine Carrington (Professor and Head of the Department of Preclinical Sciences of the Faculty of Medical Sciences at UWI-St. Augustine Campus, and GVN Affiliate Director) delivered a workshop seminar on tracking viruses in real-time and was awarded the inaugural Excellence in Regional Leadership Award for her contributions to the field and in regional capacity building. Dr. Carrington spearheaded the COVID-19 IMPACT project, establishing local whole-genome sequencing capacity for SARS-CoV-2 and providing genomic surveillance for 17 Caribbean nations during a critical window before broader regional infrastructure was available [54].

## 13. Conclusions

The 2025 GVN Regional Meeting in Jamaica was a valuable convening of arbovirology experts in the region to stimulate collaborations, highlight cutting-edge findings, and nurture the next generation of virologists. This conference brought together leading experts in arbovirology from surveillance to mitigation efforts to advance pandemic preparedness. The emphasis on Jamaica’s role in addressing arboviral threats was underscored by key lectures and workshops. The GVN remains steadfast in enhancing international partnerships, supporting leading virology research, and fostering pandemic preparedness solutions. Discussions will continue at the 2026 GVN Annual Meeting to be held at USF 4–6 March 2026, in Tampa, FL, USA.
